# Visual Interhemispheric and Striate-Extrastriate Cortical Connections in the Rabbit: A Multiple Tracer Study

**DOI:** 10.1155/2015/591245

**Published:** 2015-09-08

**Authors:** Adrian K. Andelin, David J. Bruning, Daniel J. Felleman, Jaime F. Olavarria

**Affiliations:** ^1^Department of Psychology and Behavior and Neuroscience Program, University of Washington, Seattle, WA 98195-1525, USA; ^2^BioReliance Corporation, Rockville, MD 20850, USA; ^3^Department of Neurobiology and Anatomy, University of Texas Medical School at Houston, Houston, TX 77030, USA

## Abstract

Previous studies in rabbits identified an array of extrastriate cortical areas anatomically connected with V1 but did not describe their internal topography. To address this issue, we injected multiple anatomical tracers into different regions in V1 of the same animal and analyzed the topography of resulting extrastriate labeled fields with reference to the patterns of callosal connections and myeloarchitecture revealed in tangential sections of the flattened cortex. Our results extend previous studies and provide further evidence that rabbit extrastriate areas resemble the visual areas in rats and mice not only in their general location with respect to V1 but also in their internal topography. Moreover, extrastriate areas in the rabbit maintain a constant relationship with myeloarchitectonic borders and features of the callosal pattern. These findings highlight the rabbit as an alternative model to rats and mice for advancing our understanding of cortical visual processing in mammals, especially for projects benefiting from a larger brain.

## 1. Introduction

An important goal of visual system studies in animals is to understand vision in humans. In past decades, emphasis has been placed on studying carnivores and nonhuman primates based on the belief that advanced species resemble humans in both behavior and brain organization more closely than less advanced species, such as rodents, whose visual system has been widely assumed to be much simpler by comparison. However, numerous studies in rats and mice have convincingly shown that the occipital cortex surrounding primary visual cortex (V1, striate cortex) is significantly more elaborate than previously thought, consisting of about a dozen of interconnected, topographically organized extrastriate visual areas [1–15]. This knowledge, together with the availability of genetic and molecular tools, especially in mice, has triggered a surge of studies using rodents as models for advancing our understanding of cortical visual processing in mammals. Indeed, recent advances include evidence that visual areas in rats [12, 16] and mice [15, 17–22] are functionally specialized and organized into processing streams that resemble the temporal and parietal parallel processing streams of primates and that progressive functional transformations along these pathways conform to general hierarchical principles. While these and other studies have also pointed out differences between rodents, carnivores, and primates [16, 22, 23], they nevertheless highlight the usefulness of rodents as models in mammalian visual research.

However, the small size of the brains in rats and especially mice presents some challenges, such as greater difficulty in locating and recording from small cortical areas. Experiments benefiting from, or requiring larger brains, such as studies using stimulating/recording microelectrode arrays to explore the potential for chronic recordings in visual cortex [24], or for restoring vision [25, 26], have often used rabbits, a Lagomorph whose brain is lissencephalic as in rats and mice but about 6 and 30 times larger than the brain of rats and mice, respectively. Rabbits have also been used in a variety of other investigations involving the visual cortex, including cross-modal [27] and MRI and fMRI studies [28], but at present limited information about the organization of visual cortex beyond V1 is available in this species [29–35]. Studies of the distribution of extrastriate fields labeled following single anatomical tracer injections into rabbit V1 reported that the arrangement of extrastriate areas connected with V1 closely resembles the arrangement of visual areas in rats and mice [36, 37] and suggested that the “rodent” visual cortical plan may be more general, encompassing species within the Lagomorphs and perhaps other orders [11, 36]. Whereas these previous anatomical studies identified extrastriate areas connected with V1, they were unable to reveal their internal topography due to the use of only single V1 tracer injections. To address this issue, in a group of albino rabbits we injected multiple distinguishable tracers into different regions of V1 in the same animal and analyzed the topography of resulting extrastriate labeled fields with reference to the patterns of callosal connections and myeloarchitecture revealed in tangential sections of the flattened cortex, an approach that has been fruitful for delineating the location and topography of extrastriate visual areas in several species [6, 7, 38]. We also revealed the patterns of callosal connections in a group of Dutch belted rabbits to investigate possible differences in the overall organization of this interhemispheric pathway between albino and pigmented strains of rabbits.

## 2. Methods

Surgery was performed in 5 albino and 5 adult Dutch belted rabbits (weighing approximately 2000–2500 g) anesthetized with ketamine hydrochloride (33 mg/kg, im) and xylazine hydrochloride (5 mg/kg, im), supplemented with atropine sulfate (0.05 mg/kg sc). Procedures followed protocols approved by the Institutional Animal Care and Use Committees.

### 2.1. Tracer Injections

To reveal the distribution of callosal connections in the right hemisphere, total volumes of about 9.0–10.0 *μ*L of a solution of horseradish peroxidase (HRP, Sigma Type VI, St. Louis, MO; 30% in saline) were injected into about 50–60 sites across occipital cortex in the left hemisphere. To reveal striate-extrastriate connections, small volumes (0.02–0.1 *μ*L) of several additional tracers were injected at different loci in striate cortex of the right hemisphere (approx. 4.0–10.0 mm from the midline; 4.5–14.0 mm posterior to bregma). These tracers included the anterograde tracer ^3^H-proline (25 *μ*Ci/*μ*L solution in saline, L-[2,3-^3^H] proline, specific activity 40.0 Ci/mmol, New England Nuclear, USA) and up to three retrogradely transported fluorescent tracers (rhodamine beads, RB, green beads, GB, LumaFluor, Naples, FL, USA, concentrated stock solution, and fast blue, FB, Sigma Co, St Louis. MO, 10% in DW). The approximate area of effective tracer uptake for restricted injections was estimated as described previously [39]. All tracers were pressure-injected through glass micropipettes (50–100 *μ*m tip diameter) using brief pressure pulses.

### 2.2. Histochemical Processing

After 3–5-day survival, animals were deeply anesthetized with pentobarbital sodium (100 mg/kg ip) and perfused through the heart with 0.9% saline followed by 4% paraformaldehyde in 0.1 M phosphate buffer (pH 7.4). The right cortical mantle was resected, flattened between glass slides, and sectioned tangentially (60 *μ*m thick sections) as described previously [39]. The rest of the brain received additional fixation and was cut into 60 *μ*m thick coronal sections. The transport of ^3^H-proline was revealed in sections processed for autoradiography [40], with an exposure time of 6 weeks at 4°C, while HRP labeling was revealed using tetramethylbenzidine as the chromogen [41]. A series of tangential sections was processed for myelin [42].

### 2.3. Data Acquisition and Analysis

Digital images of the myelin- and HRP-labeling patterns were obtained by scanning photographs of the histological sections at 2400 dpi using an Epson 4990 scanner. The distribution of cells labeled with fluorescent tracers was acquired with a custom, computer-assisted microscope system. Tangential sections throughout the depth of the cortex were examined to ensure that injections analyzed were restricted to grey matter.

**Figure 1:**
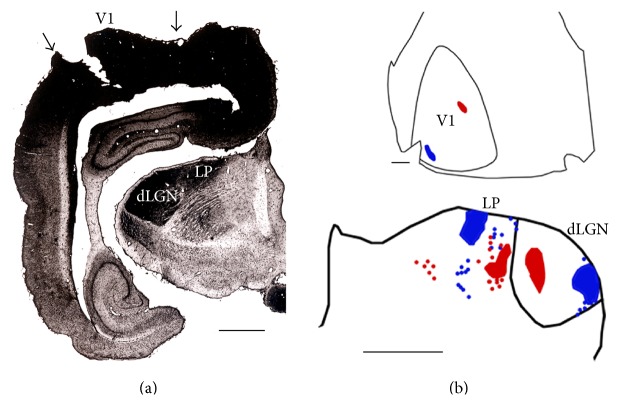
Cortical injections of HRP and fluorescent tracers. (a) Areas stained with HRP reaction product following multiple cortical injections of this enzyme. Arrows indicate borders of V1. The dLGN and LP are densely and homogeneously labeled by the transport of HRP (from case albino 86-21, Figure 5). Lateral is to the left and ventral is down. (b) Topographic retrograde labeling in dLGN and LP (lower panel) following injections of FB (blue) and red beads into V1 (upper panel) (from case albino 86-20, Figure 8). Lateral is to the right and ventral is down. dLGN: dorsal lateral geniculate nucleus, LP: lateroposterior nucleus of the thalamus. Sale bars = 2 mm.

The left hemispheres were extensively infiltrated with HRP and the ipsilateral visual thalamic nuclei were uniformly and densely labeled with reaction product (Figure 1(a)), indicating that HRP was effectively transported from the injected cortex. In the right hemispheres, the locations of the injection sites into V1 [29, 35] were confirmed by analyzing the distribution of labeled fields within the ipsilateral dorsal lateral geniculate nucleus of the thalamus (dLGN) [43] (Figure 1(b)).

Using Adobe Photoshop CS2 (Adobe Systems), digitized images of anatomical tracers and myelin labeling patterns from the same animal were carefully aligned with each other using the border of V1, the edges of the sections, blood vessels, and other fiducial marks. Cells labeled by the injections of RB, GB, and FB were represented by red, green, and blue dots, respectively, and overall labeling patterns were reconstructed from 3-4 sections. ^3^H-proline labeling was visualized under dark field microscopy and represented by outlines of densely labeled regions. Callosal connections were illustrated either by photographic images, by outlines of the areas containing dense accumulations of HRP labeling, or by thresholded versions prepared after first applying a median filter to reduce noise, followed by a high-pass filter to remove gradual changes in staining density across the entire image. The results were carefully inspected to confirm that these versions accurately represented the labeling pattern observed in the sections. Figures were prepared using Adobe Photoshop CS2, and all imaging processing used, including contrast enhancement and intensity level adjustments, was applied to the entire images.

## 3. Results

### 3.1. Myeloarchitecture

**Figure 2:**
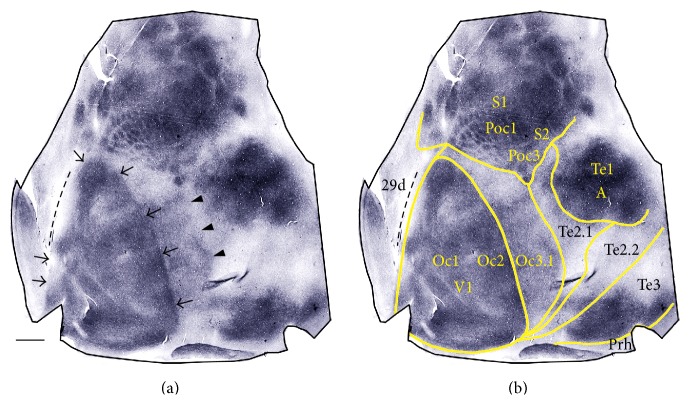
Myeloarchitectonic and cytoarchitectonic borders. Case albino 86-20. (a) Cortical myeloarchitecture. Arrows and arrowheads indicate areas of dense myelination. (b) Relation of myelination pattern to cytoarchitectonic map [44]. Segmented line indicates the lateral sulcus. Medial is to the left and posterior is down. Oc: area occipitalis, Te: area temporalis, Prh: area perirhinalis, Poc: area postcentralis, 29d: cingulate area 29d [46], S1: primary somatosensory cortex, S2: secondary somatosensory cortex, and A: auditory cortex. Scale bar = 2 mm.

Heavily myelinated areas are observed in occipital, temporal, and parietal regions (Figure 2(a)).  Figure 2(b) relates the myelination pattern to the cytoarchitectonic map of the rabbit cortex [44]. Striate cortex (area V1) appears as an oval region of dense myelination with sharp and smooth borders (Figure 2(a)). The medial border of V1 coincides with the lateral sulcus (Figure 2). Fleischhauer et al. [44] subdivided V1 into a lateral region (Oc2), which is binocular [30, 35], and a medial monocular region (Oc1), but the border between these subdivisions is not as apparent in the myelin pattern (Figure 2) as it is in other species (e.g., squirrel [45]). Area Oc3.1, immediately lateral to V1, includes the physiologically defined area V2 [30]. Area Oc3.1 is less densely myelinated than V1 but more myelinated than neighboring temporal areas Te2.1 and Te2.2. In parietal cortex, heavily myelinated regions include Poc1 (the barrel field), the rest of primary somatosensory cortex (S1), and Poc3 (second somatosensory cortex, S2). In temporal cortex, densely myelinated regions include primary auditory cortex (Te1) and a region within the most posterior and ventral portion of temporal cortex (Te3) (Figure 2(b)). In medial extrastriate cortex, the border between cytoarchitectonic areas Oc3.2 and Rsg*β* [44] was not obvious in myelin stained tangential section. We therefore identified this region as cingulate area 29d [46].

### 3.2. Pattern of Callosal Connections

**Figure 3:**
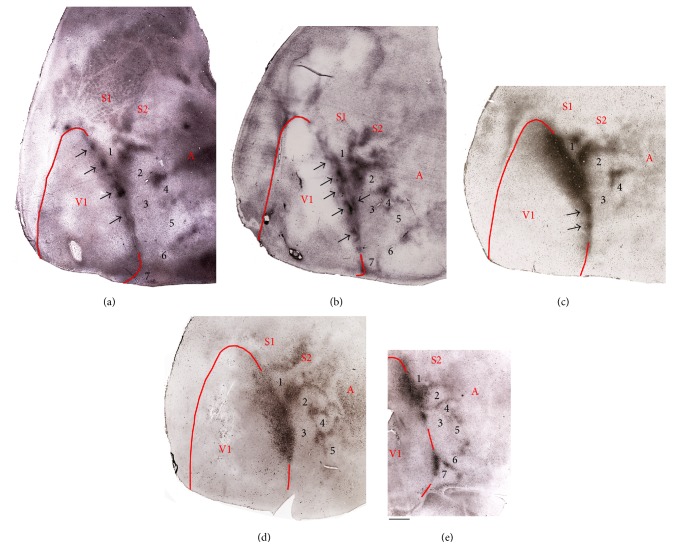
Patterns of callosal connections in the right hemisphere revealed following multiple injections of HRP into the opposite hemisphere. ((a) and (b)) Albino rabbits (cases 86-12 and 87-1, resp.). ((c)–(e)) Dutch belted rabbits (cases R53, R5, and R6, resp.). Arrows indicate bead-like patches. Opposing arrows in (b) illustrate a double patch. Numbers indicate corresponding areas in the five cases shown. Medial is to the left and posterior is down. Scale bar = 2 mm.

The patterns of callosal connections were revealed in albino (Figures 3(a) and 3(b)) and Dutch belted rabbits (Figures 3(c)–3(e)). Dense accumulations of retrogradely HRP-labeled cell bodies and anterogradely labeled terminations formed a band straddling the lateral border of V1. Often, this band had a beaded appearance, consisting of a series of distinct patches of about 0.75 mm in diameter, separated by about 1.5 mm (arrows in Figures 3(a)–3(c)). In some cases, patches were present throughout most of the lateral border of V1 (Figures 3(a) and 3(b)), while in other cases they were apparent only in posterior regions of V1 (Figure 3(c)). At some places, pairs of patches (opposing arrows in Figure 3(b)) were separated by a narrow region of low labeling density centered at the lateral border of striate cortex. The callosal band at the lateral border of V1 was consistently narrow posteriorly (0.7–1.0 mm in width), but it often tended to increase in width in more anterior regions of V1, reaching about 2.5 mm at its widest region in some cases (Figure 3(c)). It is unlikely that the patchiness and anteroposterior difference in width are due to incomplete infiltration of HRP because HRP labeling in the ipsilateral dLGN was uniformly dense throughout the nucleus (Figure 1(a)).

In extrastriate cortex callosal connections formed patches and bands, and in some cases callosal bands partially or completely encircled areas devoid of callosal connections in lateral extrastriate cortex (Figure 3). The appearance and location of several of these callosal features were consistent across rabbits and proved useful when comparing patterns of striate-extrastriate connections from different animals. No major differences between albino and pigmented rabbits were observed in the distribution of callosal connections (Figure 3).

### 3.3. Striate-Extrastriate Connections

**Figure 4:**
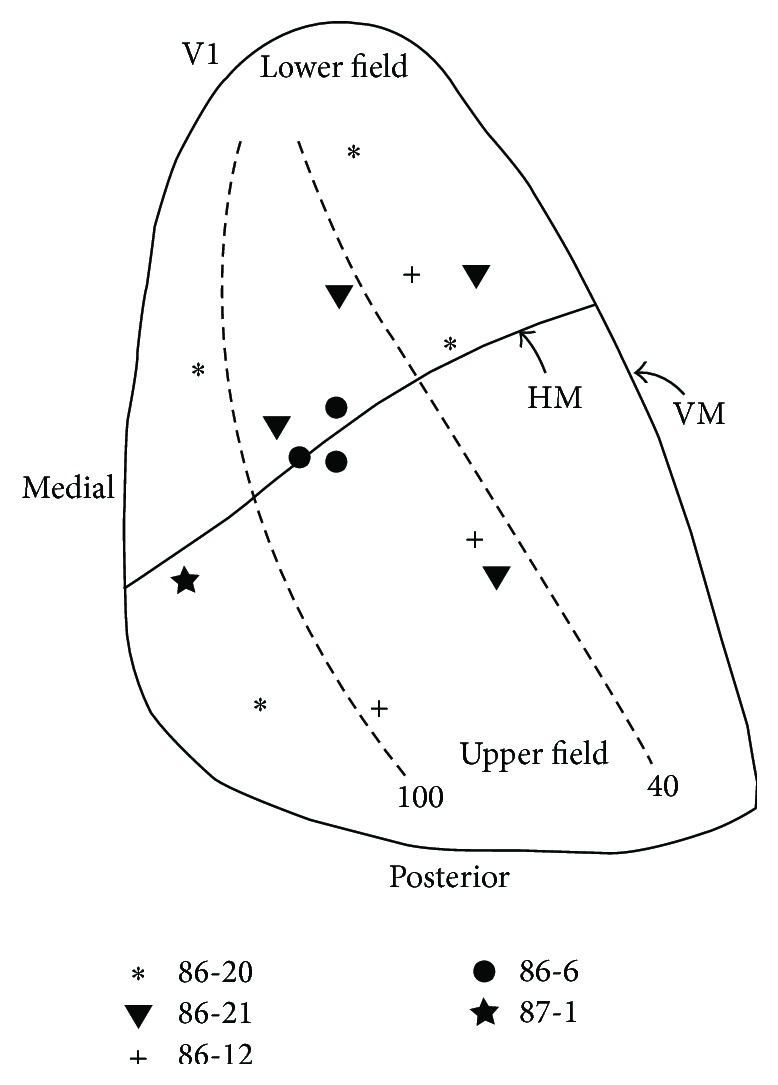
Schematic map of the visual field in right rabbit V1 [35] showing the approximate locations within V1 of 14 tracer injections performed in 5 albino rabbits. The representation of the horizontal meridian is approximate. All injections in the same animal are represented by the symbol next to the respective case number. The tracers used in each animal are indicated in the text. HM: horizontal meridian, VM: vertical meridian.

Striate-extrastriate connections were studied only in albino rabbits (*n* = 5). Tracer injections into V1 were placed in regions representing upper and lower visual fields, as well as nasal and temporal fields (Figure 4). Labeled fields of different sizes were observed in lateral extrastriate cortex. The largest fields were typically arranged anteroposteriorly forming a first tier consisting of 4-5 fields located immediately lateral to V1, in area Oc3.1, while 2-3 smaller fields formed a second tier distributed more laterally in Oc3.1 and in neighboring temporal areas Te2.1, Te2.2, and Te3. In medial extrastriate cortex, within area 29d, labeled cells usually formed an elongated field. We identified putative retinotopically-organized areas based on the analysis of local, systematic displacements of labeled fields in response to displacements of injection sites in V1. As in previous studies in the rabbit [36, 37], we have tentatively adopted the nomenclature established in the rat, in which visual areas are named according to their location relative to V1 [1, 6].

**Figure 5:**
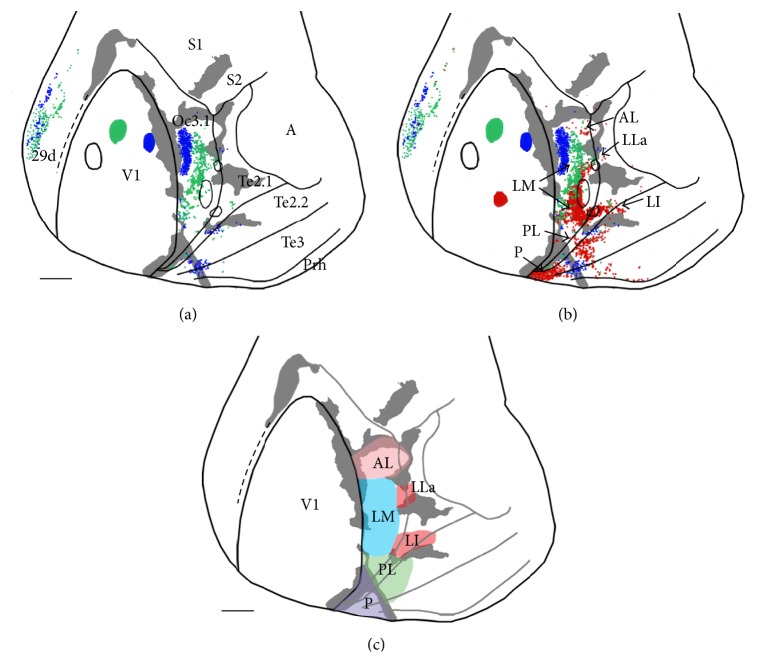
Data from case 86-21. (a) Labeling pattern following a mediolateral sequence of tritiated proline (black outlines), GB (green), and FB (blue) injections across V1. Callosal connections are indicated in grey. (b) Addition of a posterior injection of RB (red) illustrates the labeling pattern resulting from an anteroposterior sequence of injections. Arrows indicate tentative visual areas. (c) Summary of tentative visual areas identified from this case. AL: anterolateral, LM: lateromedial, PL: posterolateral, P: posterior, LLa: laterolateral anterior, and LI: laterointermediate. Scale bars = 2 mm.

The case shown in Figures 5(a) and 5(b) illustrates the distribution of extrastriate labeled fields resulting from injection sequences oriented along either the mediolateral or anteroposterior axes in V1. From these data we tentatively identified several visual areas in lateral extrastriate, which are summarized in Figure 5(c).  Figure 5(a) illustrates that a mediolateral sequence injections across the width of V1 (from temporal to nasal representations in the lower visual field) result in a mirror image distribution of labeled fields in area Oc3.1. The FB- and GB-labeled fields are elongated anteroposteriorly, occupying portions of areas that we identify as areas AL (anterolateral) and LM (lateromedial) by their locations relative to V1 and the callosal pattern (Figure 5(c)). The largest field of tritiated proline is restricted to area LM, while the two more lateral smaller fields of tritiated proline suggest the existence of additional areas lateral to LM. This animal also received an injection of RB more posteriorly in V1 (Figure 5(b)). Figure 5(b) shows a sparse field of RB-labeled cells in anterior portions of area AL and a robust elongated field in posterior portions of putative area LM, whose position was more posterior than the fields produced by the other 3 anterior injections. Further posteriorly, a callosal band extends across areas Te2.2 and Te3 in a lateroposterior direction separating two additional RB-labeled fields and partially overlapping with a FB-labeled field. The location and topography of the areas separated by this callosal band suggest that they correspond to areas PL (posterolateral) and P (posterior) described in the rat and mice [6, 7] (Figures 5(b) and 5(c)). The field of FB-labeled cells overlapping the callosal band is assumed to straddle the border between PL and P (Figures 5(b) and 5(c)).

Note that in area AL, the fields of FB- and GB-labeled cells occupy more posterior portions than the field of RB-labeled cells, and the reverse is true in LM; namely, FB- and GB-labeled cells occupy anterior regions of LM while RB-labeled cells occupy posterior regions of this area. As a result, while a region free of RB-labeled cells separates the fields of RB-labeled cells in AL and LM, the fields of FB- and GB-labeled cells appear to form continuous fields extending from anterior LM to posterior AL. In area PL, the distribution of RB- and FB-labeled cells reverses with respect to the distribution in area LM; that is, FB-labeled cells are posterior, while RB-labeled cell are anterior. Thus, as the injection site in V1 moves from anterior to posterior, the labeled fields move anteriorly in AL and PL and posteriorly in LM, suggesting that, as in the rat, the anteroposterior axis in V1 (from lower to upper visual field representations) maps along the same direction in LM but in the reverse direction in areas AL and PL. The anteroposterior map in PL appears to reverse again in area P.

The RB-labeled field in LM extends further laterally at both its anterior and posterior ends forming two tongues. The posterior tongue is long, extending into areas Te2.1 and Te2.2 (field labeled LI, laterointermediate, Figures 5(b) and 5(c)), while the anterior tongue is short with only a few RB-labeled cells seen beyond the lateral border of area Oc3.1 (field labeled LLa, laterolateral anterior, Figures 5(b) and 5(c)). Note that both of these tongues of RB labeling are located between patches of callosal connections and overlap with the two small lateral fields of tritiated proline, providing support to the idea that these two regions may correspond to two separate visual areas. Sparse RB labeling was observed in area Te3. Finally, labeling in medial area 29d suggests a mirror image representation of the mediolateral axis in V1, but RB labeling was not strong enough in this region to map the anteroposterior axis in V1.

**Figure 6:**
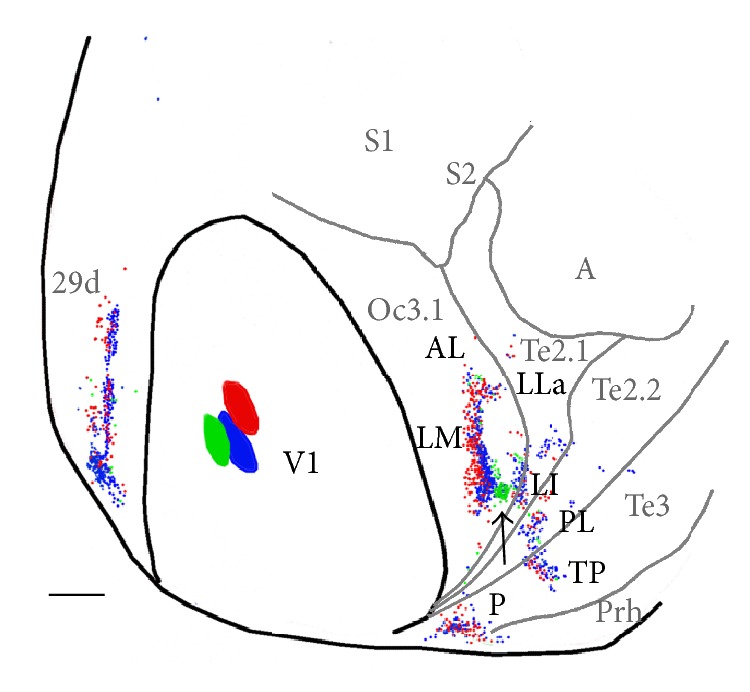
Labeling pattern produced by three closely spaced injections of RB, GB, and FB (case 86-6). A mirror-image reversal of the RB-FB-GB labeling sequence (arrow) suggests the location of the LM/LI border. Medial is to the left and posterior is down. TP: temporal posterior area. Scale bar 2 = mm.

Figure 6 provides further evidence that a mediolateral injection sequence (GB-FB-RB) produces a mirror image sequence of labeled fields (RB-FB-GB) in LM. Moreover, this sequence reverses again (GB-FB-RB) as FB-labeled cells and RB-labeled cells are found immediately lateral to the GB-labeled field (indicated by an arrow). This small, labeled field straddles the Oc3.1/Te2.1 border and extends into Te2.1. It appears to correspond to the area occupied by both the posterior isotope-labeled field and the posterior tongue-like RB-labeled field described in Figure 5(b). Together, these data provide further evidence that this region may correspond to area LI in the rat, in which the mediolateral map reverses with respect to that in LM [47]. Thus, in Figure 6, the GB-labeled field indicated by the arrow (temporal visual field representation) would mark both the lateral border of LM and the medial border of LI. More posteriorly, Figure 6 shows separate labeled fields that may correspond to areas PL and P described above. While distribution of RB-and FB-labeled cells in LP suggests a mirror image of the corresponding injection sites in V1, the topography in P is not clear. Immediately lateral and posterior to LP, in area Te3, we tentatively identify area TP (temporal posterior area, see below). Labeling could not be assigned with certainty to area AL. In medial extrastriate cortex, a narrow field of labeling was oriented anteroposteriorly, but its topography was not apparent.

**Figure 7:**
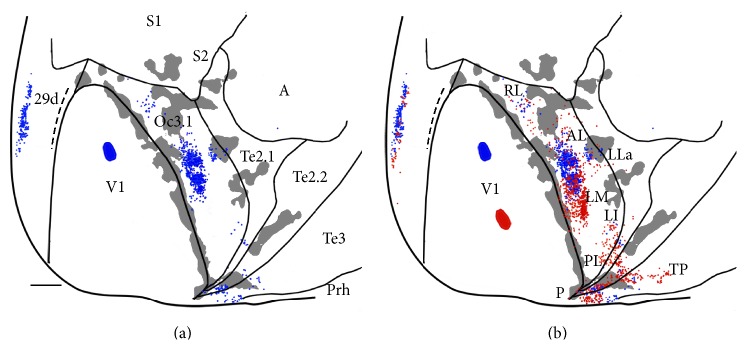
Data from case 86-12. ((a) and (b)) Labeling pattern resulting from an anteroposterior sequence of FB and RB injections. Callosal pattern (shown in Figure 3(a)) is indicated in grey. Medial is to the left and posterior is down. Scale bar = 2 mm.

**Figure 8:**
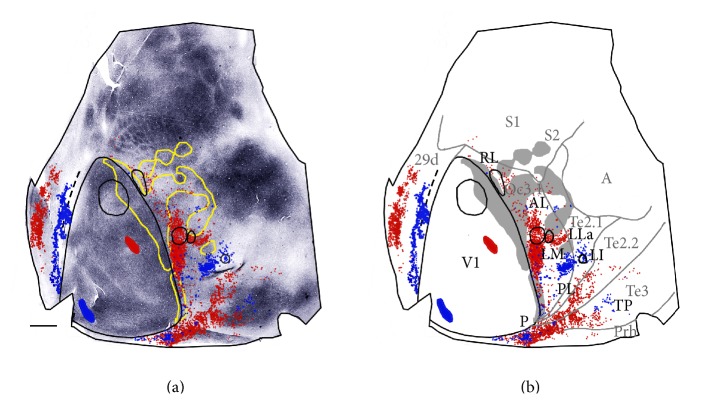
Data from case 86-20. (a) The myelin pattern (also shown in Figure 2(a)) is related to the callosal pattern (yellow contour) and to labeling resulting from injections of tritiated proline, RB, and FB. (b) The location of putative visual areas is indicated. Callosal pattern is represented in grey. Medial is to the left and posterior is down. Scale bar = 2 mm.

Additional data on the mapping of the anteroposterior axis in V1 are presented in Figures 7 and 8. Following an injection of FB in a region of V1 representing lower visual fields, two dense, slightly separated FB-labeled projection fields were observed lateral to V1, in regions likely corresponding to areas AL and LM (Figure 7(a)). Comparing these data with data from an injection of RB placed more posteriorly, in regions representing upper fields (Figure 7(b)), shows that the distribution of RB-labeled cells is more extensive, occupying more anterior regions in AL as well as more posterior regions in LM. These displacements in opposite direction are consistent with data in Figure 5 suggesting that the anteroposterior map in V1 has the same orientation in LM but is inverted in AL. A small field of FB- and RB-labeled cells is observed lateral to the larger RB- and FB-labeled fields associated with areas AL and LM. This small field appears to be in a region corresponding to area LLa, and the fact that FB-labeled cells are located more anterior than the RB-labeled cells suggests that the anteroposterior axis in V1 is mapped along the same orientation in area LLa. Further posteriorly, this case shows labeled fields resembling those seen in Figures 5 and 6, in regions that appear to correspond to areas LI, PL, P, and TP. Anterior to AL, there was sparse FB and RB labeling in a narrow region delimited laterally by a callosal band. This region may correspond to area RL (rostrolateral, Figure 7(b)) described in the rat [12, 47] and mouse [14]. Labeling similar to that described in Figures 5 and 6 was observed in medial extrastriate cortex.

Figure 8(a) correlates the pattern of callosal connections (outlined in yellow) and the labeled fields resulting from injections of tritiated proline (black outlines), RB, and FB with the underlying myeloarchitecture. The injection of tritiated proline in anterior V1 (lower visual fields) produced a circular labeled field in area Oc3.1, which likely straddles the border between areas AL and LM. A smaller labeled field is located immediately lateral, in an area we tentatively identify as area LLa (Figure 8(b)). Another isotope-labeled field was observed anteriorly in area Oc3.1, in a region we identify as area RL. This region is less densely myelinated than the rest of area Oc3.1. The injection of RB was placed more posteriorly in V1 and, consistent with the topography of areas AL and LM described above, the RB-labeled fields in AL and LM appear to fuse at the site of the large isotope-labeled fields, presumably at the border between AL and LM, where the representations of lower visual fields in AL and LM meet. However, unlike this isotope-labeled field, RB-labeled fields extended more anteriorly in AL and more posteriorly in LM, occupying regions that represent higher elevations. A tongue-like RB-labeled field extended laterally between callosally labeled regions. This field overlapped with the small, lateral isotope-labeled field, supporting the existence of a small area we call LLa in this region (see Figures 5(b) and 7(b)). In putative area RL, the RB labeling was located further lateral than the isotope labeling, suggesting that RL is topographically organized. In posterior regions, the RB injection produced labeled fields in areas Te2.2 and Te3 (Figure 8(b)) that resemble the labeling localized in areas PL, P, and TP in Figures 5, 6, and 7. The RB- and FB-labeled fields in area TP occupy a portion of a densely myelinated area observed in Te3 (Figure 8(a)), and the separation between these fields suggests that area TP is topographically organized. Labeling in this region was observed following injections into different V1 sites, suggesting that area TP represents a large portion of the visual field. The injection of FB was placed very close to the posterior and medial border of V1, in a region representing peripheral portions of the upper visual field. In lateral extrastriate cortex, FB-labeled cells accumulated in lateral area Oc3.1, in approximately the same location occupied by the GB-labeled field in Figure 6 (black arrow), and by the two more posterior isotope-labeled fields in Figure 5(b). These results support the interpretation drawn from Figures 5(b) and 6 that peripheral visual fields are represented at the boundary between putative areas LM and LI. A small FB-labeled field was located at the posterior end of lateral extrastriate cortex, likely in area P, and a few FB-labeled cells were located in area AL. In medial extrastriate cortex, elongated FB- and RB-labeled fields overlapped extensively in the anteroposterior direction, while along the mediolateral axis they were segregated, suggesting a mirror-image representation of the mediolateral axis in V1.

In a final case (87-1) we revealed the projections from an injection of tritiated proline placed at the medial border of posterior V1, in a region roughly similar to the FB injection in Figure 8. We observed a labeling pattern resembling that produced by the FB injection in Figure 8 (data not shown). In both cases the distribution of labeling in lateral extrastriate cortex was rather restricted, suggesting that extreme upper peripheral visual fields are not represented in all extrastriate areas.

## 4. Discussion

### 4.1. Callosal Connections

We confirmed studies showing that callosal connections form a band that straddles the lateral border of V1 [48, 49]. Our results extend these previous observations by showing that in lateral extrastriate cortex of both albino and pigmented rabbits callosal connections form patches and several band-like regions oriented mediolaterally at different anteroposterior levels. In some cases, a band of callosal labeling was observed in anterior portions of medial extrastriate cortex. The location of several of these callosal features was constant across animals of both strains. However, we did not typically observe ring-like callosal configurations encircling separate extrastriate cortical regions, as described in rodents [6] and some marsupials [50, 51].

The callosal band at the V1/Oc3.1 border often had a beaded appearance in both albino and Dutch belted rabbits. The presence of callosal patches along this band is in agreement with previous reports in rabbits [52] and squirrels [53]. A recent study in Long Evans rats correlated distinct periodicities in the pattern of callosal connections in V1 with ipsilateral ocular dominance columns [54], but a similar correlation may not exist in rabbits and squirrels because no evidence of ocular dominance columns has been found in these species [55, 56]. It remains possible that callosal projection patches at the V1 border in rabbits and squirrels relate to orientation selectivity or other forms of functional segregation [57].

Relative to the width of V1, the callosal zone in V1 is narrower in rabbits than in rats [30, 58]. A possible explanation comes from relating the width of the binocular regions in V1 and the projections from temporal retina in both species. The binocular region in rabbit V1 [30, 59] is narrower than in rats and other species, reflecting the fact that the rabbit temporal retina is relatively small due to the more lateral placement of the eyes [35]. Moreover, as in the rat [60], the entire temporal retina of rabbits projects both ipsilaterally and contralaterally [61]. Consistent with the hypothesis that the width of the callosal zone in V1 reflects the extent of temporal retina from which crossed projections originate [62–64], the width of the callosal zone in V1 matches the width of the binocular region in V1 in both rats [54] and rabbits [30, 59]. Thus, relative to the width of V1, the difference in the width of the V1 callosal zone between rabbits and rats may simply reflect the difference in the width of the binocular zone between these species. It is worth adding that both the width of the binocular region in rabbit V1 [29, 30] and the strength of the ipsilateral eye input to this region [65] tend to decrease posteriorly, which may explain our observation that the width of the callosal zone in rabbit V1 tends to decrease posteriorly.

### 4.2. Striate-Extrastriate Connections

**Figure 9:**
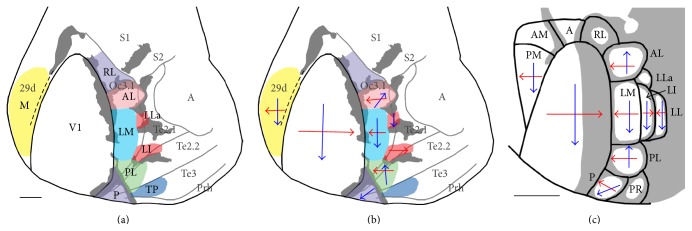
Tentative diagram of rabbit visual areas and topography derived from present study, and comparison with the rat. ((a) and (b)) The callosal pattern (grey) is based on case 86-21 (Figure 5). (a) Location and name of putative visual areas. M: medial area. (b) Approximate representations in extrastriate areas of the mediolateral (red arrows) and anteroposterior (blue arrows) axes in V1. Lack of arrows indicates insufficient data. (c) Diagram of rat visual areas based on physiological and anatomical studies [1–6, 9–12, 16, 36, 39, 47, 70]. Callosal pattern is represented in grey. A: anterior area, AM: anteromedial area, PM: posteromedial area, and PR: pararhinal area. Scale bars = 2 mm.

Our results extend previous studies and provide further evidence that extrastriate areas identified anatomically in the rabbit [36, 37] resemble the pattern of visual areas in the rat not only in their general location with respect to V1 but also in their internal topography. Our data are also consistent with the interpretation that, as in the rat, lateral extrastriate areas connected with V1 are arranged primarily in two tiers. Figures 9(a) and 9(b) show a tentative diagram of the distribution and internal topography of extrastriate visual areas derived from this study. To facilitate comparison with studies in rodents, a diagram of visual areas in the rat is illustrated in Figure 9(c). The anteroposterior and mediolateral oriented arrows (Figure 9(b)) summarize the displacements of the injections sites in V1 and the resulting displacements of labeled fields in some of the identified extrastriate areas. Most injections into rabbit V1 labeled fields widely distributed in the areas delineated in Figures 9(a) and 9(b), with the exception of very medial and posterior injections (see Figure 8). The latter injections produced more restricted labeling patterns, suggesting that only some extrastriate areas contain representations of extreme upper and temporal regions of the visual field. Representations that are either incomplete or biased toward particular regions of visual space have also been described in mice [15].

In rats (Figure 9(c)) and mice, areas AL, LM, PL, and P form a tier located adjacent to the lateral border of V1. In these areas, the representation of the mediolateral axis in V1 (from temporal to nasal visual fields) is inverted, such that tracer injections into medial or lateral regions of V1 produce labeled fields away or close to the lateral border of V1 in lateral extrastriate cortex, respectively [1–17, 47]. We observed a similar topography in the homonymous areas in the rabbit. The similarity extended to the representation of the anteroposterior axis in V1 (from lower to upper visual fields). As in the rat and mice, we observed that the anteroposterior axis in V1 maps along the same direction in area LM but in the reverse direction in both AL and PL. Our data (Figure 5) also suggest that the map reverses again in P. Opposite orientations of the elevation maps in LM and AL are illustrated by the fact that labeled fields in LM and AL originating from progressively more anterior loci in V1 (representing progressively lower visual fields) moved closer and closer together, eventually merging. As such, the most anterior V1 injection results in a single field at the putative border between AL and LM (see isotope labeled field in Figure 8(b)). We also identified area RL whose location in anterior lateral extrastriate cortex resembles that of area RL in rats [12, 47] and mice [14]. In these rodents, area RL is often associated with a small anterior callosal ring, and in some rabbits callosal connections form a ring-like configuration in this region (Figure 3).

In agreement with Montero [36], we observed that area LM is the largest area in lateral extrastriate cortex and that it is elongated in the anteroposterior direction. On the basis of its location, size, and topographic organization, we concur with Montero's suggestion that LM corresponds to an area called V2 in previous electrophysiological studies in the rabbit [30, 31, 35] and that it is likely homologous to visual area V2 described in primates, carnivores, and other species [66].

In addition to the first tier of areas located immediately adjacent to the lateral border of V1, we identified a second tier of smaller areas. In one of these, area LI, the mediolateral topography was a mirror image of that in LM, as in rats [6, 9, 11, 16] and mice [7, 14]. A small area called LL has been identified further laterally in rats [6, 9, 11, 16] and mice [7], in which the mediolateral topography reverses again, resembling the map in LM. While next to LI we may have failed to identify an area corresponding to area LL in rats and mice, we tentatively named LLa a small area we identified further anteriorly (Figure 9(a)). The second tier is somewhat variable in the rat, and in some studies area LLa has been identified anterior to LL [6, 47]. Thus, it is possible that area LLa in the rabbit may correspond to area LLa in the rat and mouse [15].

The densely myelinated area we observed in Te3 may correspond to a heavily myelinated area, called TP (temporal posterior), described in approximately this region in the squirrel [67, 68] and agouti [69]. The portion connected with V1 may correspond to a visually responsive area in rabbit temporal cortex described in previous physiological and anatomical studies [33, 34]. In the rat, it may correspond to a region connected with V1 described in perirhinal cortex [6, 11, 12] (see area PR, pararhinal, in Figure 9(c)) and to the caudal temporal area [70], while in the mouse it may correspond to area 36p [14].

We did not observe projections anterior to V1 that could correspond to area A (anterior) in rats [6] and mice [14]. Likewise, we did not observe projections to a site in somatosensory cortex called S in rats [6] and mice [7]. Medially, in area 29d, we observed connections with an elongated region that we tentatively called area M (medial, Figure 9(a)). Labeling from different injections usually overlapped extensively, but in some animals the anteroposterior axis in V1 was represented as in V1, while the mediolateral axis in V1 was represented as a mirror image. This region may correspond in part to area AM described in the rabbit [36] and areas PM, AM, and M described in rats [6, 9, 11] and mice [7, 14, 15, 18]. More detailed experiments will be necessary to correlate labeling in medial cortex with the architectonic subdivisions recognized by Fleischhauer et al. [44] in this region.

In addition to areas V1 and V2, a previous physiological study [31] described three small visual areas in regions corresponding approximately to areas AL and LLa in the present study. Further posteriorly, the same study described an additional small area in regions that may correspond to area PL or TP in this study. These findings support the notion that areas identified anatomically in the rabbit likely correspond to separate representations of the visual field, as it has been demonstrated in the rat and mice. Additional studies combining electrophysiological and anatomical methods will be required to further explore the topography and interconnectivity of the areas identified in this and previous anatomical studies [36, 37].

## 5. Conclusion

Our study provides further information about the location and topography of extrastriate areas connected with V1 in the rabbit and relates these areas to the patterns of callosal connections and myeloarchitecture. Our results should facilitate the interpretation of additional mapping and hodological data obtained in the rabbit with electrophysiological and other techniques and contribute toward comparative studies of the organization of visual cortex in mammals. In view of the similarity that appears to exist between rabbits, rats, and mice, the rabbit offers an alternative model for further studies of the “rodent” visual cortical plan, especially for projects benefiting from a larger brain.
